# Supplementary material for: Association Between Working Memory at Age 4 Years and Night Sleep Duration and Yogurt Intake Frequency at Age 1 Year
Source: Nutrients. 2025 Sep 27;17(19):3081. doi: 10.3390/nu17193081 (PMC12526159; doi:10.3390/nu17193081)
Supplement: Supplementary file 1 [file nutrients-17-03081-s001.zip › nutrients-3870647-supplementary.pdf]

Table S1. Spearman correlations between 1-year-olds' yogurt intake frequency and sleep measures.

	**1-year-olds' frequency of yogurt intake (points)**
1-year-olds' total sleep duration (hours)	−0.06
1-year-olds' night sleep duration (hours)	0.02
1-year-olds' day sleep duration (hours)	−0.05
1.5-year-olds' total sleep duration (hours)	−0.06
1.5-year-olds' night sleep duration (hours)	0.03
1.5-year-olds' day sleep duration (hours)	−0.11
3-year-olds' total sleep duration (hours)	0.01
3-year-olds' night sleep duration (hours)	−0.10
3-year-olds' day sleep duration (hours)	0.08
